# FDG-PET vs. chemical shift MR imaging in differentiating intertrabecular metastasis from hematopoietic bone marrow hyperplasia

**DOI:** 10.1007/s11604-021-01149-x

**Published:** 2021-06-08

**Authors:** Nozomi Oki, Yohei Ikebe, Hirofumi Koike, Reiko Ideguchi, Daisuke Niino, Masataka Uetani

**Affiliations:** 1 Department of Radiological Sciences, Nagasaki University Graduate School of Biomedical Sciences, 1-7-1 Sakamoto, Nagasaki, 852-8501 Japan; 2 Department of Diagnostic and Interventional Radiology, Hokkaido University Hospital, N14, W5, Kita-ku, Sapporo, 060-8648 Japan; 3 Department of Radioisotope Medicine, Atomic Bomb Disease Institute, Nagasaki University, 1-12-4 Sakamoto, Nagasaki, 852-8523 Japan; 4 Pathology, University of Occupational and Environmental Health, 1-1 Iseigaoka, Yahatanishi-ku, Kitakyushu, 807-8555 Japan

**Keywords:** Intertrabecular metastasis, Hematopoietic bone marrow hyperplasia, FDG-PET CT, MRI, Chemical shift imaging

## Abstract

**Purpose:**

To evaluate the utility of SUVmax on FDG-PET and chemical shift imaging (CSI) on MRI in the differentiation of intertrabecular metastasis (ITM) from hematopoietic bone marrow hyperplasia (HBMH).

**Patients and methods:**

We retrospectively evaluated 54 indeterminate focal bone marrow lesions in 44 patients detected on FDG-PET. The lesions were assigned to the metastasis group (M group, 29 lesions of 24 patients) and the non-metastasis group (non-M group, 25 lesions of 20 patients) based on the follow-up or the histopathological studies. The lesions were assessed with the maximum standardized uptake value (SUV_max_) on FDG-PET CT images and signal change ratio (SCR) on CSI.

**Results:**

The median SUV_max_ were 5.62 and 2.91; the median SCR were − 0.08 and − 34.8 in M and non-M groups respectively, with significant difference (*p* < 0.001). With ROC curve analysis, the optimal cutoff value of SUV_max_ was 4.48 with a sensitivity of 72.4%, a specificity of 100%, and AUC of 0.905. The cutoff value of SCR was − 6.15 with a sensitivity of 82.8%, a specificity of 80%, and AUC of 0.818.

**Conclusion:**

FDG-PET and CSI on MRI are useful in distinguishing ITM from HBMH. Though their sensitivities are similar, the specificity of FDG-PET was higher than that of MRI.

## Introduction

Bone metastasis is classified into four types on the basis of the accompanying bone responses: osteolytic, osteoblastic, mixed, and intertrabecular types. Intertrabecular metastasis (ITM) is characterized by tumor growth without significant trabecular bone changes. Actually, this type of bone metastasis is most common, reportedly accounting for approximately 37% of all cases of metastatic bone lesions at autopsy; it is often found in cases of small-cell lung carcinoma, hepatocellular carcinoma, and hematological malignancies [1]. ITM can be easily missed in the clinical setting because they are often negative on conventional radiographs, computed tomography (CT) or bone scintigraphy. Magnetic resonance imaging (MRI) has been reported to be reliable to detect ITM [2]. Furthermore, a meta-analysis shows ^18^F-fluoro-2-deoxy-d-glucose PET (FDG-PET) has a superior diagnostic ability for metastasis from lung cancer than MRI and bone scintigraphy [3]. However, there have been no studies focusing on the diagnostic performance of FDG-PET in detecting ITM.

ITM presents high accumulation on FDG-PET and abnormal signal on MRI: low signal on T1-weighted imaging (T1WI) and high signal on fat-suppressed T2-weighted imaging (T2WI) or short tau inversion recovery (STIR) sequences. Those findings are nonspecific when there are no structural bone changes. Therefore, ITM needs to be differentiated from various bone marrow lesions which shows abnormal findings in FDG-PET or MRI.

Hematopoietic bone marrow hyperplasia (HBMH) is a variation of bone marrow with the proliferation of hematopoietic cells presenting as local or diffuse “indeterminate skeletal lesion”, which shows accumulation on PET and abnormal signal on MRI [4, 5]. It is commonly associated with heavy smoking, long-distance running, obesity, granulocyte colony-stimulating factor (GCSF) administration as adjuncts to radiation or chemotherapy and severe anemia. It is also common in individuals with malignant tumors, in whom differential diagnosis from bone metastasis is particularly important.

As HBMH contains fat as well as hematopoietic cells, detection of a fatty component can be a clue in differentiating HBMH from intertrabecular metastasis. Previous studies showed that HBMH showed higher signal intensity than intervertebral disks or skeletal muscles on T1WI reflecting its fatty component; however, the results depended on visual assessment without objective criteria [6, 7]. Chemical shift imaging with the use of in-phase (IP) and opposed phase (OP) imaging is a special MR technique to detect a fatty component mixed with a water component. Hematopoietic marrow is supposed to show signal loss on OP images because of the presence of both fat (fatty marrow) and water (hematopoietic cells). The utility of CSI for the differentiation of benign and malignant bone lesions in the spine has been reported [4, 8–11]. However, these studies included a variety of bone or bone marrow lesions and there have been virtually no studies which investigated the role of CSI for the diagnosis of indeterminate skeletal lesions, such as HBMH or intertrabecular metastasis.

Focal HBMH shows high accumulation on FDG-PET [5, 12]. In one study, the maximum standard uptake value (SUV_max_) of HBMH was significantly lower than that of bone metastasis. However, the number of cases was limited, and cases of bone metastasis were not restricted to intertrabecular metastasis. No reliable cutoff value of SUV_max_ has not been yet available to differentiate intertrabecular metastasis from HBMH.

The purpose of the study is to evaluate the utility of FDG-PET CT and CSI in the differentiation of ITM from HBMH.

## Patients and methods

The clinical research institutional review board of our institution approved this study.

### Patient selection

**Fig. 1:**
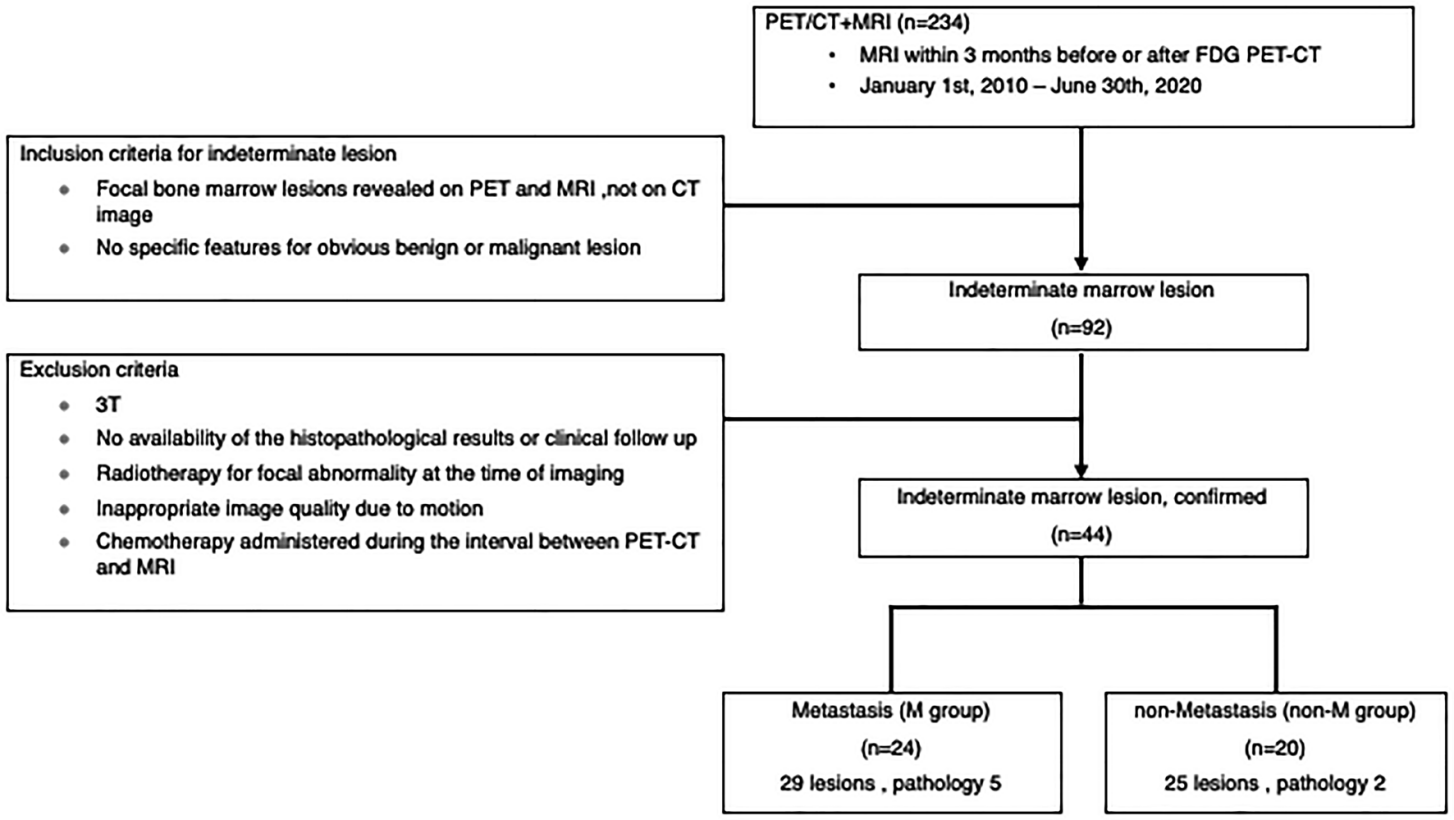
Flowchart showing eligibility criteria and the resulting number of study patients

We searched our radiological reports from January 2010 to June 2020, using the keywords “bone metastasis,” “hypercellular marrow,” and “red marrow”, and selected 234 patients who underwent MRI with CSI on the bone and soft tissue within three months after ^18^F-FDG-PET CT. Upon the consensus of two radiologists with expertise in bone and soft tissue imaging, images were reviewed without the final diagnosis disclosed, and 92 patients were selected based on the following criteria: (1) localized bone marrow lesions on PET–CT and MRI and no clear abnormality on CT and (2) no definite morphological findings suggesting malignant or benign lesions. Among the 92 patients, 48 were excluded due to the following reasons: 3 T MRI, no confirmed diagnoses based on histopathology or no clinical follow-up, chemotherapy administered during the interval between PET-CT and MRI, and inappropriate imaging quality due to motion. We finally selected 54 lesions of 44 patients of indeterminate bone marrow lesions (Fig. 1). The patients comprised of 30 men and 14 women with a median age of 63 years (range 10–90 years).

The final diagnoses of these indeterminate bone marrow lesions were decided based on the follow-up studies at least 6 months from the initial imaging study or confirmed by the histopathological studies. In follow-up studies, enlargement of the lesions, the appearance of bone destruction, sclerotic change or extraosseous mass on clinical imaging were considered to be the findings of malignancy, and lesions that did not present any interval changes were considered benign. Finally, the lesions were assigned to metastasis group (M group) and non-metastasis group (non-M group). The M group consisted of 29 lesions of 24 patients where five lesions had pathological diagnoses and 24 were determined in the follow-up studies. As osteolytic and osteoblastic metastases were excluded, these lesions were likely intertrabecular metastasis. The non-M group consisted of 25 lesions of 20 patients, where two lesions were diagnosed pathologically as HBMH and 23 were diagnosed based on the clinical course as probable HBMH.

### MRI and FDG-PET protocols

MR imaging was performed on 1.5T MR imaging scanners (GoldSeal Signa HDxt; GE Healthcare, Waukesha, WI, USA and MAGNETOM Avanto, Siemens Healthcare, Erlangen, Germany). In addition to CSI (OP and IP gradient-echo images), T1-weighted turbo spin echo (TSE) images, T2-weighted TSE images, STIR or fat-suppressed (FS) T2WIs were obtained. CSI parameters are summarized in Table 1.

**Table 1:** MR chemical shift imaging acquisition parameters

	GE	Siemens
Repetition time (ms)	175–200	140
Echo time (ms)	4.5–4.9/2.2–2.5	4.76/2.38
Flip angle (°)	80	75–85
No. of slices	13–24, 72	11–28
Section thickness/gap (mm)	3–6/0.5–1.5	3–5/0.5–1.5
Matrix	256 × 224–288 × 224	256 × 154–448 × 336
Field of view (cm)	24–36	24–36

In whole-body FDG-PET CT, data were acquired with a dedicated combined FDG-PET CT system (Biograph mCT; Siemens Healthcare, Erlangen, Germany). Patients fasted for 6 h before scanning. The median of blood glucose level was 108 mg/dl (84–237). An hour before imaging, 141.4–303.1 MBq (median: 223 MBq) of 18F-FDG were injected intravenously. We obtained unenhanced CT images before PET with a 16-slice helical scanner (tube voltage 120 kV, Quality ref. mAs 80, rotation time 0.5 s, pitch 0.8, collimation 16 × 1.2 mm). Then we obtained whole-body PET images covering the skull base to the midthigh level.

### Image analysis

MRI and FDG-PET CT were analyzed using a picture archiving and communication system (PACS) (SYNAPSE, FUJIFILM, Japan). Lesions were identified by carefully comparing their anatomical localization on both modalities. In cases involving multiple lesions, lesion with the highest FDG accumulation was selected for analysis. The maximum diameter was measured in the cross-sectional images of MRI on either T1-weighted, STIR or opposed phase images.

Circular regions of interest (ROI) were set for the target lesions to measure the maximum standardized uptake value (SUV_max_) on FDG-PET CT images. On MRI, circular ROIs were placed in the same sites as those of FDG-PET CT, which were as large as possible within the lesions on both in-phase (IP) and opposed-phase (OP) images. Subsequently, the signal change ratio (SCR) of OP relative to IP was measured according to the following formula:

$${\text{SCR}}\, = \,\left[ {\left( {{\text{SI OP}}{-}{\text{SI IP}}} \right)/{\text{SI IP}}} \right]\, \times \,{1}00 \, \left( \% \right),$$

where SI represents signal intensity.

### Statistical analysis

The reproducibility of SUV_max_ and SCR measurements was determined by examining the intra-examiner and inter-examiner reliability using the interclass correlation coefficient.

The median SUV_max_ and SCR of the M and non-M groups were compared using the nonparametric Mann–Whitney *U* test. To assess the diagnostic performance of SUV_max_ and SCR to differentiate the two groups, we obtained receiver operating characteristic (ROC) curves, and calculated the sensitivity and specificity based on the optimal cutoff value of SUV_max_ and SCR.

In all tests, we used *p* values of < 0.05 to denote statistical significance. The statistical software used was JMP (SAS Institute, Cary, North Carolina, USA).

### Results

Clinical characteristics of patients in M and non-M groups including age, sex, types of malignancy, and sites of the lesions are summarized in Table 2. No significant differences in age, red blood cell count, hemoglobin level, and hematocrit level were noted between the groups. The maximum diameter of the lesions on MRI was 22.6 mm (range 9.5–138.8 mm) in the M group and 26.5 mm (range 15.3–96.6 mm) in the non-M group, not indicating any significant difference.

**Table 2:** Clinical characteristics among the studied group of patients

	M group (*n* = 24)	Non-M group (*n* = 20)
Sex M/F	14/10	16/4
Age (years)^a^	62.5 (10–81)	66 (40–90)
Number of lesions	29	25
History of malignancy (*n*)	24	18
Malignant lymphoma	7	1
Head and neck cancer	3	2
Lung cancer	4	3
Breast cancer	3	0
Gastrointestinal cancer	3	8
Others	4	5
Location of bone marrow lesion		
Spine (cervical/thoracic/lumbar /sacrum)	2/3/6/1	0/7/2/4
Pelvic bone (ileum/pubis/ischium)	6/1/0	4/0/2
Femur	6	6
Others	Scapula (1), humerus (3)	0
RBC (10^4^/µL)^a^	432 (327–533)	401 (310–482)
Hb (g/dL)^a^	12.2 (9.3–15.9)	13.0 (8.9–15.3)
Hct (%)^a^	37.5 (29.4–47.0)	38.9 (30.0–45.8)
History of chemotherapy (*n*)	7	3
History of GCSF administration (*n*)	0	0
MR unit (GE/Siemens)	4/20	6/17

^a^Data are the median (min. to max.)

**Fig. 2:**
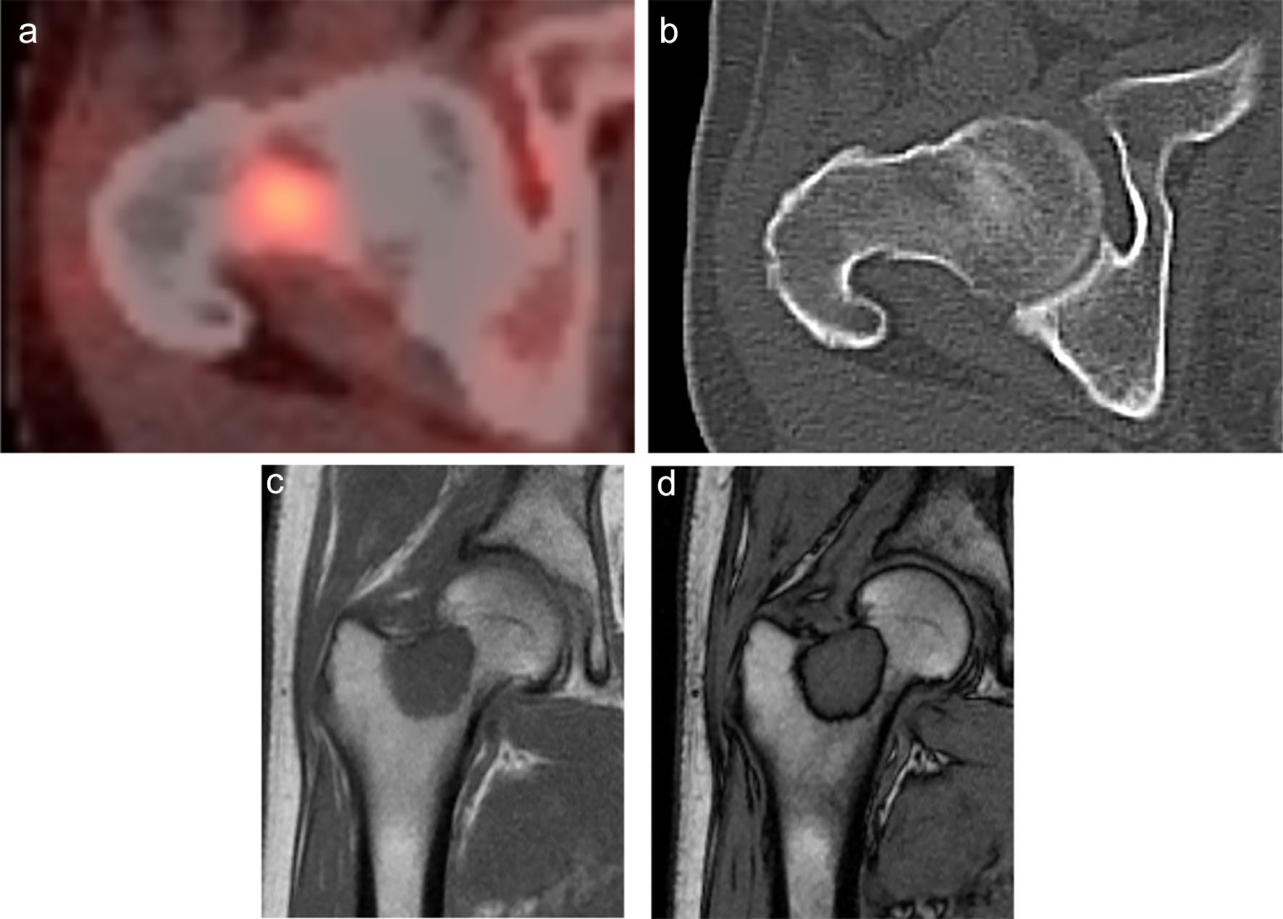
A 65-year-old male patient with a history of lung cancer. FDG-PET CT image **a** shows a focal hot spot in the right femur. CT finding is almost normal but maybe slightly osteoblastic in retrospective interpretation (**b**). SUV_max_ of the right femur lesion is 4.8. Coronal in-phase **c** demonstrates a signal intensity of 100.4 whilst the opposed-phase **d** shows a signal intensity of 100.3. Signal change ratio (SCR) on CSI was − 0.08%, compatible with a malignant lesion. Histopathological study revealed metastatic lung cancer

**Fig. 3:**
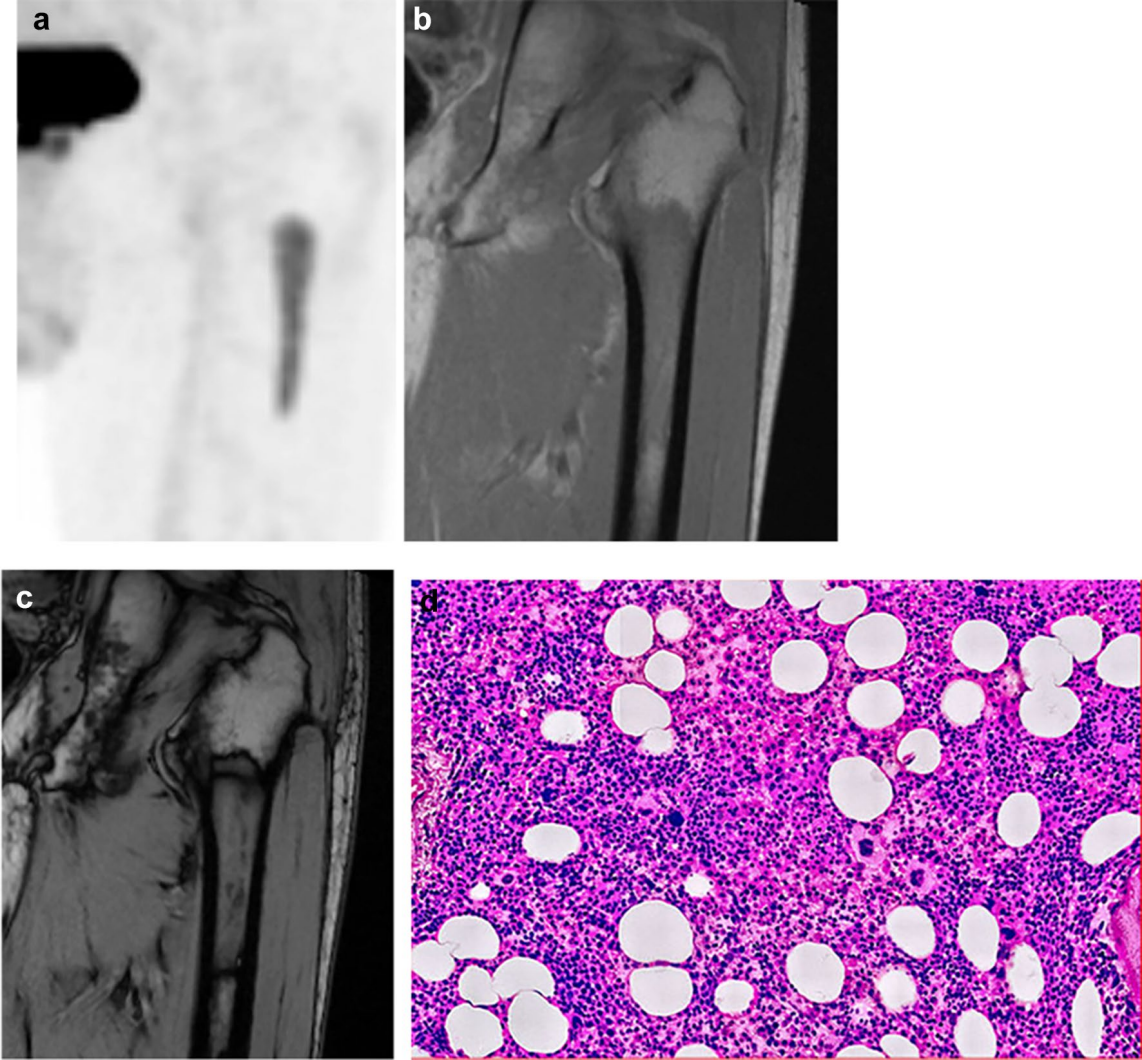
A 69-year-old male patient with no history of malignancy. Coronal FDG-PET CT image **a** shows a focal hot spot in the left femur. SUV_max_ of the left femur lesion is 4.0. Coronal in-phase **b** demonstrates a signal intensity of 175.1 whilst the opposed-phase **c** shows a signal intensity of 155.4. Signal change ratio (SCR) on CSI wa﻿s − 11.2%, compatible with a benign lesion. Biopsy was performed to exclude the possibility of malignant lesion, which revealed hyperplastic hematopoietic bone marrow (**d**)

**Fig. 4:**
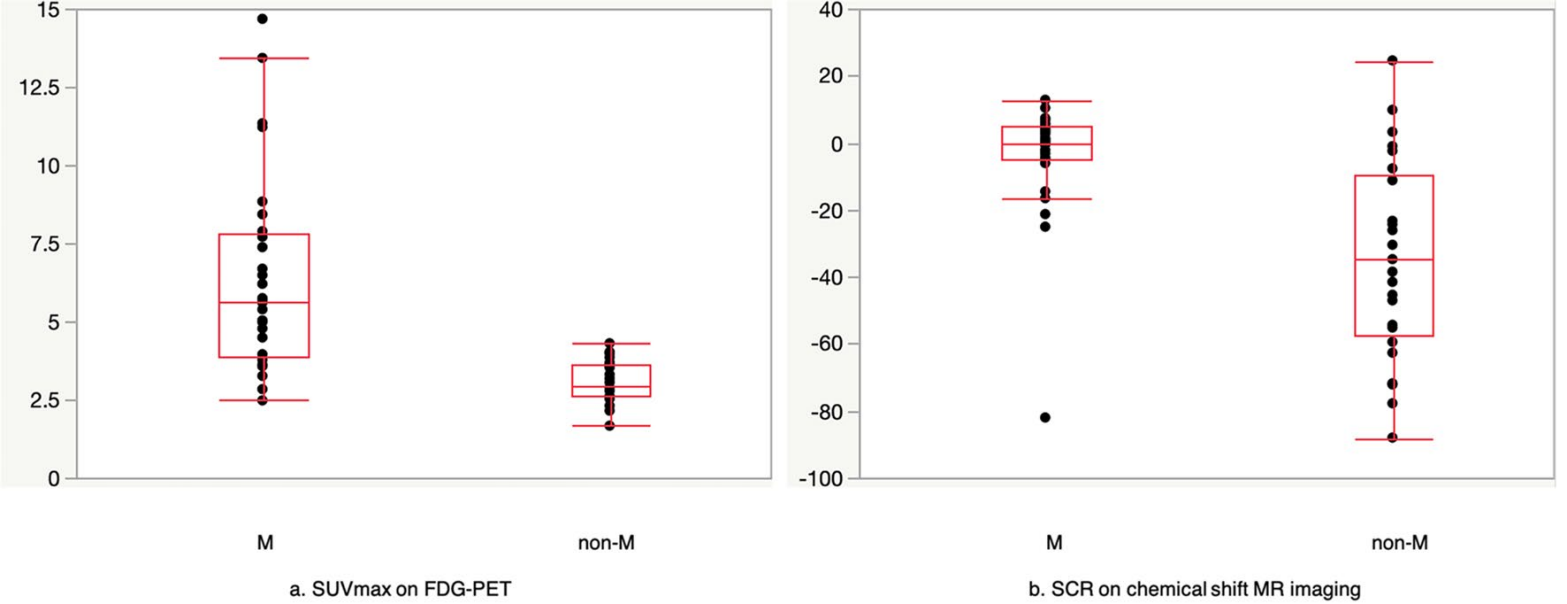
SUV_max_ on FDG-PET CT (**a**) and signal change ratio (SCR) on chemical shift MR imaging (**b**). Significant difference was found between M group and non-M group in both parameters

**Fig. 5:**
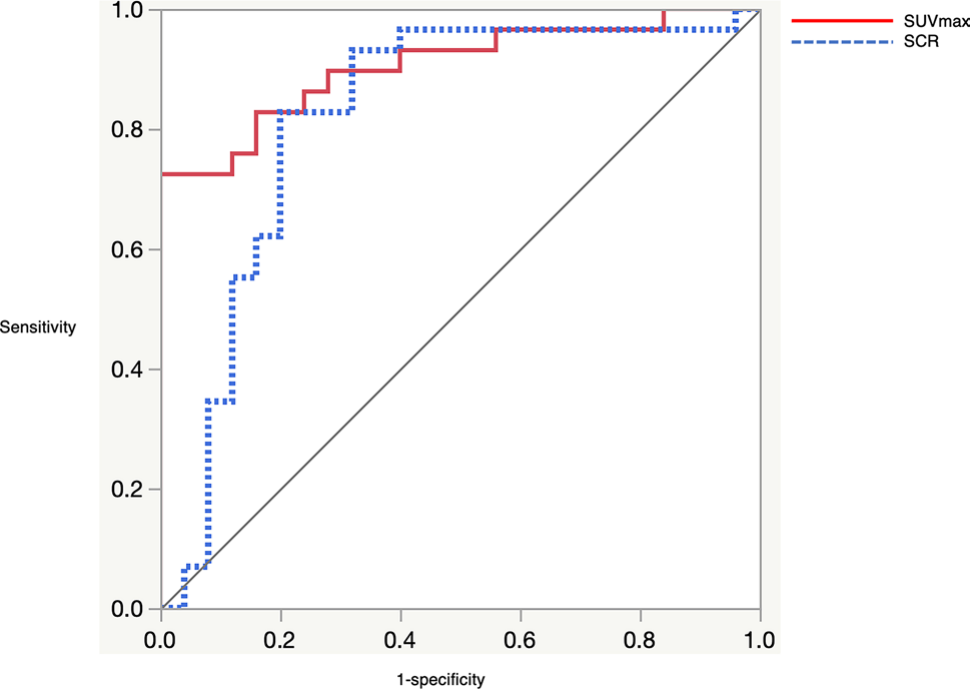
ROC curves of imaging parameters. AUC for SUV_max_ is 0.905 (95% CI 0.788–0.961), AUC for signal change ratio (SCR) is 0.818 (95% CI 0.655–0.914)

Figures 2 and 3 show representative cases of ITM and HBMH, respectively, which were correctly differentiated based on both SUV_max_ of FDG-PET CT and SCR of CSI. Figure 4 summarizes the results of SUV_max_ and SCR of both groups. The median SUV_max_ in M and non-M groups were 5.62 and 2.91, respectively. No significant correlation was found between the maximum size of the lesions and the above parameters in both groups. The median SCR in M and non-M groups were − 0.08 and − 34.8, respectively. These values were significantly different in both groups (*p* < 0.001). With ROC curve analysis (Fig. 5), the optimal cutoff value of SUV_max_ was 4.48 with a sensitivity of 72.4%, a specificity of 100%, and AUC of 0.905. The cutoff value of SCR was − 6.15 with a sensitivity of 82.8%, a specificity of 80%, and AUC of 0.818.

Eight false-negative lesions with the cutoff SUV_max_ value of 4.48 were observed.

**Fig. 6:**
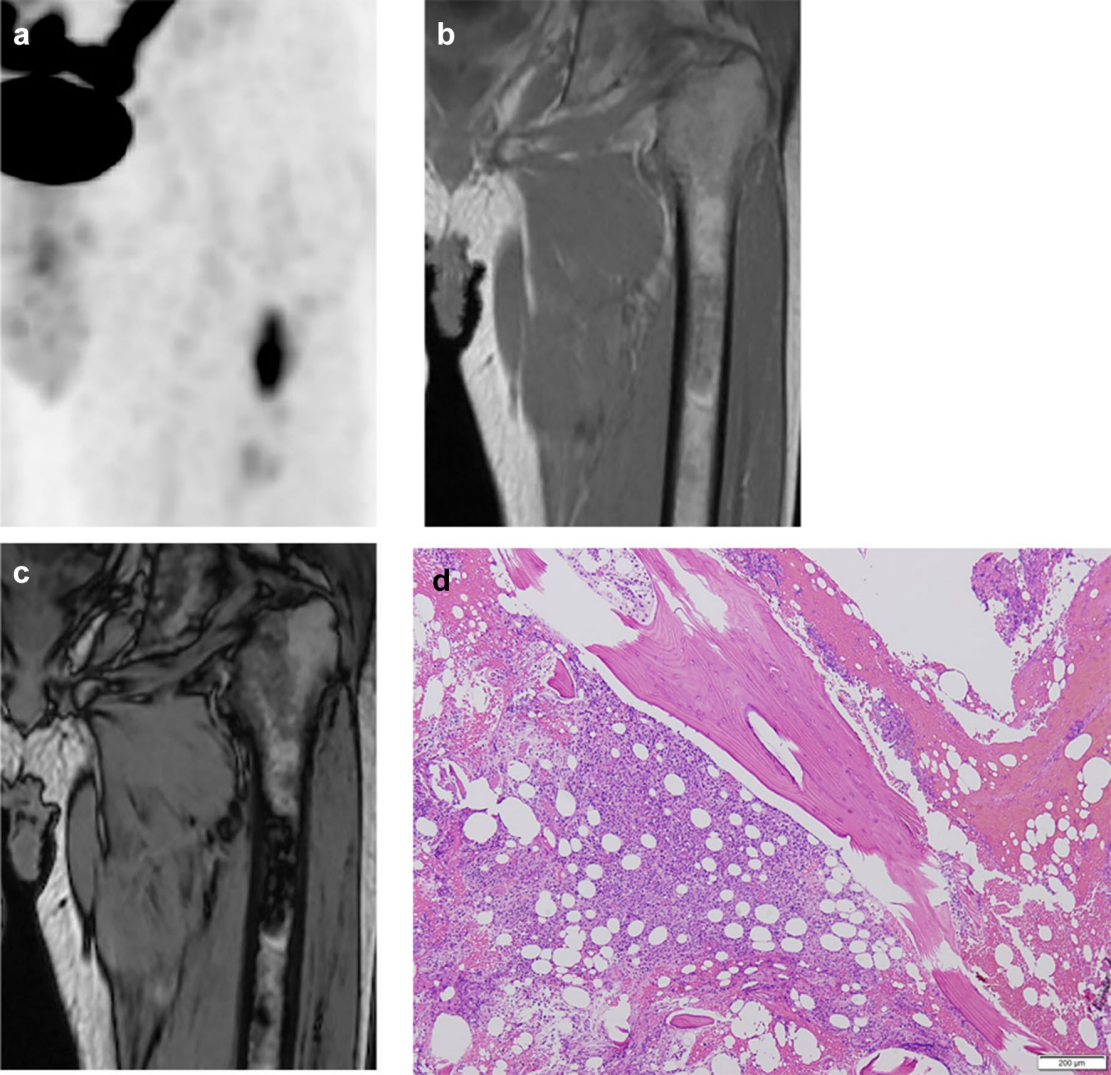
A 65-year-old male patient with a history of malignant lymphoma in clinical remission. Maximum intensity projection of FDG-PET CT image **a** shows a focal hot spot in the left femur. SUV_max_ of the lesion is 7.7, which is high enough to suggest metastasis. Coronal in-phase **b** demonstrates a signal intensity of 300.1 whilst the opposed-phase **c** shows a signal intensity of 53.8. Signal change ratio (SCR) on CSI was −﻿ 82.1%, suggesting a content of fat in the lesion. Biopsy of this lesion showed metastasis of large cell lymphoma (**d**). The histologic picture shows tumors cells infiltrating into the medullary space with preservation of bone marrow fat. The signal loss on opposed-phase images, a false negative result, can be explained by the content of fat

Five false-positive and five false-negative lesions were observed with the cutoff SCR value of − 6.15. Two false-negative lesions were pathologically proven as recurrent malignant lymphoma (Fig. 6) and metastasis of rectal cancer, where both lesions contained residual bone marrow fat intermingled with neoplastic tumor cells.

### Intra-examiner and inter-examiner reliability

Regarding intra-examiner reliability, the interclass correlation coefficient was 0.9987 for SUV_max_ and 0.975 for SCR. Regarding inter-examiner reliability, the interclass correlation coefficient was 0.9998 for SUV_max_ and 0.8367 for SCR, thus indicating a high degree of consistency.

## Discussion

This study examined the efficacy of FDG-PET CT and MRI in differentiating ITM from non-metastatic lesions in patients with bone marrow lesions where abnormal accumulation was indicated on FDG-PET CT. Non-metastatic bone lesions targeted for differentiation in this study were limited to bone marrow lesions without bone changes, and most of them were likely HBMH. These metastatic and nonmetastatic bone marrow lesions showed similar signal intensity patterns compared with adjacent bone marrow on conventional MRI, including T1WI, T2WI and STIR.

The cutoff value for SUV_max_ on PET–CT to differentiate the two conditions was 4.48 with a sensitivity of 72.4%, a specificity of 100%, and an accuracy of 85.2%. Histological differentiation, cell density, and the size of the lesion likely affect the accumulation of FDG, but the cause for false negatives or false negatives could not be determined. In a previous study by Shigematsu, et al., imaging findings of 8 cases of pathologically confirmed HBMH of the vertebral body were compared with those of spinal metastasis [5]. The results of their study suggested SUV_max_ > 3.6 for differentiating metastasis from HBMH; however, the sample size was small (8 cases of hyperplastic bone marrow and 24 cases of bone metastasis), hence with no statistical analysis. The metastatic lesions of their study were not restricted to ITM. It is, therefore, difficult to compare the results of their study with ours.

Suh et al. [13] conducted a meta-analysis of the diagnostic ability of CSI for differentiating benign and malignant vertebral marrow lesions (12 studies including 663 lesions in 591 patients), which indicated a pooled sensitivity of 0.92, a pooled specificity of 0.89, and hierarchical summary receiver operating characteristic (HSROC) AUC of 0.95. In these studies, the cutoff value of the signal ratio (OP/IP) and the rate of signal decrease in OP were 0.8–1 and 1.44–35%, respectively. However, the previous studies included benign bone marrow lesions other than HBMH, such as benign bone tumors, fractures, spondylosis or spondylitis. Furthermore, bone metastases were not restricted to ITM. As the present study targeted cases that only presented abnormal signals of the bone marrow without bony morphological changes, it is difficult to directly compare the results of the present study with those of existing studies regarding diagnostic abilities.

Two lesions of malignant lymphoma were included in five false-negative cases. Based on the study by Arbur et al. [14], bone marrow infiltration by lymphoma can be pathologically divided into diffuse, nodular, paratrabecular, interstitial, and mixed patterns; biopsies performed on patients with non-Hodgkin lymphoma most frequently showed mixed pattern(46.4%), followed by paratrabecular (15.8%), nodular (15.6%), diffuse (12.9%), and interstitial (9.3%). In cases of diffuse infiltration, normal hematopoietic and fat cells are almost replaced by tumor cells, but in other types, fat cells tend to remain in the bone marrow. Such a form of bone marrow infiltration by malignant lymphoma may be the cause for the false-positive results in CSI. In fact, the histopathological studies of the present study showed residual bone marrow fat in the false-positive cases of malignant lymphoma. Other infiltrating bone marrow tumors, such as multiple myeloma, or tumors containing fat, such as metastatic renal cell cancer can show an increase of SCR [15].

False-positive cases in CSI, which showed reduced or no signal loss in OP, can be associated with fibrosis, bleeding due to fracture or magnetic susceptibility artifact caused by osteosclerosis or hemosiderin deposition [15]. Since the present study targeted lesions without bone changes, fracture or osteosclerosis can be ignored, but the possibility of fibrosis or hemosiderin deposition cannot be excluded. Signal loss in OP can be also affected by the degree of hematopoietic hyperplasia; when bone marrow fat is almost replaced by hematopoietic cells, it may lead to a false-positive result.

Recently, an MR technique for quantifying fat fraction (FF) in tissues (proton density fat fraction [PDFF]) using modified Dixon sequence became available. By using chemical shift-encoded MRI where confounding factors such as T1 effects, T2* effects, the presence of multiple peaks in the fat spectrum or eddy current effect, are corrected; then, FF in tissues can be more correctly measured than chemical shift imaging. Kim et al. measured FF on three-echo VIBE Dixon sequence, lesion-disc ratio on T1-weighted sequences and contrast-enhancement ratio on pre- and post-gadolinium enhanced fat-suppressed T1-weighted images for 21 malignant bone marrow lesions and 11 benign red marrow depositions and examined their diagnostic abilities [16]. The median value for FF was 12.8% for malignancy and 37.3% for benign red marrow depositions, indicating a statistically significant difference. When 16.8% or below was considered the cutoff value of malignancy, the sensitivity was 85.7%, specificity was 100%, and the AUC was 0.961, which were superior to those of other parameters. In this study, the morphology of bone metastasis was variable, but benign red marrow deposition was selected as a control group similar to the present study. It is difficult to make a general comparison of their result with ours, but it is reasonable that FF has a better diagnostic ability than CSI in differentiating bone metastasis from red marrow, because magnetic susceptibility effect due to bony trabeculae can be reduced by T2* correction. Further study is required to compare the diagnostic performance of FF measurement and CSI in distinguishing benign and malignant bone marrow lesions.

When we encounter bone marrow lesions in patients with or without malignancy, pathological confirmation by bone biopsy is often needed to make an immediate decision on treatment [17–19]. In a study by Barbara Raphael et al., the biopsy was performed on bone lesions found in 482 patients with known primary malignancy; the results showed that 316 cases (66%) had metastasis from the known primary lesion, 15 cases (3%) had metastasis from a new primary lesion, and 103 cases (21%) had benign lesions. Half of the benign lesions were normal bone marrows (54 cases) (21), which could include hematopoietic marrow hyperplasia. By proper use of FDG-PET and MRI, we can avoid such unnecessary biopsies. In that sense, specificity may be more important than sensitivity. On the other hand, better sensitivity should be emphasized to avoid missing metastasis. The cut-off value should be applied carefully considering the clinical situation and purpose of the studies.

Our study had several limitations. First, the sample size was small, which might have led to a bias in the results. Second, this study adopted a retrospective study design. Third, many lesions were not pathologically confirmed, because it is ethically difficult to perform a biopsy when the lesions showed imaging findings with a high likelihood of malignancy or benign lesions. Thus, most of the lesions were determined on the follow-up studies of six months or more. Fourth, although care was taken to draw the ROI on the lesions, the study results could be dependent on readers’ decisions. However, Intra-examiner and inter-examiner reliability were fair in measurement. Fifth, MRI machines from two different vendors were used. A study stated that vendors and the magnetic field strength of MR units did not lead to a significant difference in signal intensity index [20]. Thus, we assume that the difference in the vendor did not have a notable impact. Finally, the images included various sites. The spine was the most common site in this study, constituting approximately 46%, followed by the pelvic bone (24%) and the femur (22%). However, we believe that the results were not significantly influenced by differences in the imaged sites.

In conclusion, both SUV_max_ in PET–CT and SCR in MRI are useful in diagnosing intertrabecular metastasis and differentiation from HBMH. Though their sensitivities are similar, the specificity of PET–CT was higher than MRI. Bone marrow fat may remain in intertrabecular metastasis, which can be a cause of false-negative results of chemical shift MR imaging.
